# What motivates or demotivates injecting drug users to participate in hypothetical HIV vaccine efficacy trials? A qualitative study from urban Tanzania

**DOI:** 10.24248/eahrj.v4i2.636

**Published:** 2020-11-26

**Authors:** Masunga K. Iseselo, Edith AM Tarimo, Eric Sandstrom, Asli Kulane

**Affiliations:** a Department of Clinical Nursing, Muhimbili University of Health and Allied Sciences, Dar es Salaam, Tanzania; b Department of Nursing Management, Muhimbili University of Health and Allied Sciences, Dar es Salaam, Tanzania; c Equity and Health Policy Research Group, Department of Global Public Health, KarolinskaInstitutet, Tomtebodavägen 18A, 171 77 Stockholm, Sweden; d Department of Clinical Science and Education, Södersjukhuset, Karolinska Institutet, Tomtebodavägen 18A, 171 77 Stockholm, Sweden

## Abstract

**Background:**

HIV vaccine efficacy trials require the active participation of volunteers who are committed and adherent to the study protocol. However, information about the influence of Injecting Drug Users (IDUs) to participate in HIV vaccine efficacy trials in low-income countries is inadequate. The present study explored the factors that motivate or hinder IDUs from participating in HIV vaccine efficacy trials in Dar es Salaam, Tanzania.

**Methods:**

A qualitative descriptive study design was employed among IDUs at Muhimbili National Hospital (MNH). A purposeful sampling technique was used to recruit the participants. Three (3) focus group discussions (FGDs) and 10 In-Depth Interviews (IDIs) were used to collect the data. The data from participants were audio-recorded, transcribed, and analysed using the content analysis approach.

**Findings:**

The participants reported that altruism and the desire to reduce risks of HIV infection were the motivators to participate in hypothetical HIV vaccine trials. In addition, participants reported to consult close relatives towards motivation to participate in the vaccine trial. In contrast, the perceived fear of vaccine side effects, lack of information about HIV vaccine studies, and HIV-related stigma towards participants were described as barriers to participate in the HIV vaccine trials.

**Conclusion:**

Participation in a hypothetical HIV vaccine trial among IDUs is influenced by positive and negative factors. Actual recruitment plans could be made through a better explanation of HIV vaccine trials, the expected individual and collective benefits associated with the trials. Community involvement and sensitisation is likely to enhance participation in future HIV vaccine trials in Tanzania.

## BACKGROUND

Since the beginning of the epidemic, injecting drug behaviours are the key facilitators of HIV transmission^1^. In 2017, 15.6 million Injecting Drug Users (IDUs) aged 15to 64 years were identified worldwide, of whom 17.8% were living with HIV^2^. A substantial geographic variation in the prevalence of HIV infection across all countries has been noted. The number of IDUs in South-East Asia and East Asia has increased^3^. Evidence shows an increase in injection drug use and associated HIV infections in Sub-Saharan Africa, the region with the highest global HIV prevalence^4,5^.

Although the overall HIV prevalence in Tanzania has decreased from 5.1% in 2012 to 4.7% in 2016^6^, the prevalence of HIV among IDUs is still high at 42%.^7^ Factors associated with an increase in HIV infection among IDUs include age, sharing of syringe and needles, low level of education, and history of drug overdose.^8–10^ Various harm reduction programs such as methadone assisted treatments have been developed as one of the strategies for combating HIV infection among IDUs.^11,12^ Additionally, the development/deployment of an Integrated Methadone and Antiretroviral Therapy (IMAT) have shown to be effective in decreasing HIV prevalence among IDUs in Tanzania.^13^ However, a multi-lateral approach including behavioural interventions, Treatment as prevention (TasP), pre-exposure prophylaxis, and vaccines is more likely to lead to effective infection control than deployment of a single approach.^14–16^

The efforts dedicated to discovering an HIV vaccine have a long history since HIV was first described in the early 1980s. The potential to slow the HIV epidemic and save lives fuels the development of an effective vaccine.^17^ Clinical trials require the active participation of volunteers who are committed and adhere to the study protocols. Vaccine efficacy trials take years to monitor the effectiveness and side effects of any new vaccine. The process is more challenging for HIV vaccine efficacy trials because it involves socio-behavioural issues that affect the volunteers’ participation.^18,19^

The successful development and implementation of a safe and efficacious vaccine greatly depend on the extent to which the at-risk populations are motivated to participate in HIV vaccine research.

Studies in high-income countries report financial reimbursement, reduction of risk behaviours, and support from researchers as the motivating factors among men who have sex with men (MSM) and female sex workers (FSW).^20–22^ However, the social pressure of significant others, perceived lack of vaccine safety, and logistical concerns have also been identified as barriers to actual participation in the trials.^23^ Also, misconceptions about HIV vaccine trials, personal and social risks, and costs are reported to hinder participation in hypothetical HIV vaccine trials in many high-income countries.^24^ However, there is a paucity of information about motivations and barriers for participating in HIV vaccine trials among injecting drug users in low-income countries.

A recent study in Kenya reported a desire to receive healthcare and information about HIV were the motivating factors to participate in an HIV vaccine trial among MSM and FSW^25^. Furthermore, a review of the literature reported that retention and sexual disinhibition were the main socio-behavioural challenges for HIV vaccine efficacy trials in Sub-Saharan Africa.^26^ However, none of these studies reported motivations and barriers to participating in HIV vaccine trials among IDUs in low-income countries. Thus, this study describes the reasons behind the willingness of IDUs to participate in the HIV vaccine trials and provides recommendations for future studies.

## MATERIALS AND METHODS

### Design

A qualitative descriptive study design was employed. We applied this approach to better understand the fundamental motives influencing participation in HIV vaccine trials among IDUs. This design was also used to raise awareness and increase insight into the best ways of conducting HIV vaccine trials in the study population.^27^ In addition, the approach allowed interaction between the authors and the participants which increased trust among each other.

### Setting

The study was conducted at the Methadone Clinic in Muhimbili National Hospital (MNH), Dar es Salaam. The site is 1 of the 3 Medication-Assisted Treatment clinics (MAT) for IDUs who are trying to stop the use of heroin in the region. The MAT clinic at MNH was the second to be opened in Sub-Saharan Africa after Mauritius^28^.

The clinic is well staffed throughout the year to accommodate IDUs to come to the clinic daily to receive a Directly Observed Dose (DOT) of liquid methadone. The maximum duration of treatment to recovery for most IDUs was 5 years, although some finished treatment in 3 years, depending on medication adherence and effective use of counselling services. The authors were not part of the MAT clinic staff and had no affiliation with the hospital where the participants were receiving treatment services.

### Population and Participants

This study was based on a high-risk population attending the MAT clinic. We selected the IDUs for their relatively high risk of HIV infection compared to other key populations in Tanzania.^29^ The risk of IDUs was largely due to a history of needle and syringe sharing practices and that was suitable for the HIV vaccine efficacy trial.

### Inclusion and Exclusion Criteria

The study included participants who were injecting drugs, physically and mentally stable, aged 18 and above, and HIV negative. Both males and females were included. IDUs who were physically and mentally unstable and those that could not communicate appropriately were excluded.

### Sampling Procedure

We used purposive sampling to recruit the participants as proposed by Palinkas et al.^30^ The decision to use this sampling technique was based on information-rich participants who could be willing to share their concepts and views about HIV vaccine studies. This sampling technique allowed the authors to obtain adequate information related to the phenomena of interest. A trained research assistant selected the participants from the MAT clinic.

### Sample Size

The sample size was determined using the principles of saturation. That is, we terminated sampling when no new information was obtained as proposed by Hennink et al.^31^

### Data Collection Briefing Sessions

Before the commencement of data collection, we provided a brief overview of HIV vaccine efficacy trials to the participants, including the nature of vaccine material, how it would be administered, what to expect if the participant is enrolled in the study, and the issues related to vaccine-induced seropositivity and how it could be handled. This information promoted awareness of HIV vaccine efficacy trials, since some participants had never heard about these trials before.

### Focus Group Discussions (FGDs)

FGDs were used as the main data collection method. We first conducted FGDs to identify the main recurrent ideas. This method provided an opportunity to interact with the participants and explore their understanding of HIV vaccine trials. The group discussions were conducted immediately after the participants had taken their daily methadone doses to ensure maximum cooperation. The group size ranged between 6 and 8 participants.

We used homogeneous FGDs in the sense that male and female groups were conducted separately (2 groups and 1 group of males and females respectively). This approach allowed the free expression of ideas and views among the participants. A discussion guide was used to collect the data. The questions in the guide comprised of core questions from previous studies.^32–34^ which were improved further through pilot testing.^35^ The questions elicited the general views about the risk of HIV infection, the level of motivation to adhere to treatment protocols, and any perceived barriers toward HIV vaccine trials.

The following questions were asked during the group discussion:

1. What are your views on the risk of HIV infection due to injecting drugs?
2. What are your views if you are asked to participate in HIV vaccine efficacy trials?
3. What would motivate you to participate in an HIV vaccine efficacy trial?
4. How would the people you live with influence your decision to participate in HIV vaccine efficacy trials?
5. What would hinder you from participating in HIV vaccine trials?

These were followed by specific probing questions to obtain additional information or clarification. The first and second authors reviewed the discussion guide after the first FGD to include more emerging themes. The FGDs lasted between 45 and 60 minutes. During the group discussion, the first author moderated the discussion while a research assistant took notes and controlled the external environment. Data saturation was reached when no new information was obtained after a new group was added as guided by Hennink et al^31^ resulting in 3 focus group discussions.

### In-Depth Interviews (IDIs)

To complement the data from FGDs, we conducted IDIs with IDUs who participated and who did not participate in the FGDs. The individual interviews were conducted in a quiet, well-lit room in the hospital premises away from the MAT Clinic. This was important to ensure safety and maximum cooperation from the participant as well as to making the environment natural.^36^

The IDIs elicited more descriptive information on HIV vaccine trial participation. The first author conducted all interviews. The IDIs used the same questions that were applied in the FGDs. However, some specific probing questions were geared towards individual participants such as: How would your schedule fit in with a proposed HIV vaccine trial? What are your views on the availability of an effective preventive HIV vaccine? After the 10^th^ IDI, we reached information saturation. Of these 10 interviews, 4 participants (3 males and 1 female) had participated in FGDs. These participants were invited to take part in the IDIs because during FGDs, the first author observed that they were hesitant to share their views; however, they appeared to have some ideas. The interviews lasted between 30 to 40 minutes. Both FGDs and IDIs were conducted in Kiswahili, the language spoken by most people in Tanzania, and was well understood by the participants. Data were audio-recorded.

### Data Analysis Focus Group Discussions

Data analysis started as soon as the first FGD was completed. This guided the subsequent levels of questions and probes in the discussion guide. The research assistant (a nurse) transcribed verbatim of all the audio-recorded data in the Kiswahili language and typed it into the Microsoft Word computer program. The first and second authors checked the transcripts against the audio-recorded data to ensure the correctness of the transcribed data. The unit of analysis was a whole transcript. All files of transcripts were transferred to NVivo 11.0 software (QSR International, Melbourne, Australia) for coding and organisation. The texts were analysed in the native language of the participants. The first 2 authors read the transcripts iteratively and thoroughly to immerse themselves in the data. Interesting content areas were coded, as guided by content analysis principles.^37^ We used inductive coding whereby codes were developed from the data using phrases or terms utilised by the participants themselves. In this way, we were able to stay close to the data, mirroring what is actually in them. The coding of the contents continued throughout the rest of the documents. Reflecting on the objective of the present study, code classifications were created containing defined attributes related to the topic of interest. After we had coded all information and organised it into a manageable format, all codes were shared between the first 2 authors for discussion, and the consensus was reached on the coded information. The process of sharing the codes helped to improve the credibility of the coding system and organisation^38^. We then continued reading and abstracting the contents into more specific ideas that were mutually exclusive of each other. In other words, the text was divided into meaning units that were condensed, abstracted, and eventually labelled with codes. Coding continued for all transcripts to form categories and themes. (Table 1).

**TABLE 1:** Example of Meaning Units, Condensed Meaning, and Codes, Category, and Theme

Meaning unit	Condensed meaning	Codes	Category	Theme
*“When I decide to participate in a vaccine trial against HIV infection, I will be helping my country. Also, if this vaccine becomes effective, it will be helpful to me, as well as other people and other countries.”*	Participating in the vaccine to help the country	Helping the country	Altruism	
	An effective vaccine will help other people	Helping other propel		
				Motivation
*“I am ready to be enrolled in a involves participant education of risk-related behaviour, study because this activity including screening for different infectious diseases. Participating in HIV vaccine trials is will give information that helps to protect me from infection”*	Being enrolled to get behaviour and health check-ups	Getting education	Desire to reduce the risk for HIV infections	
		Health screening		
	Participation helps protect from infection	Protecting from infection		

### In-Depth Interviews

The same analysis process was carried out on the IDIs data. Following the analysis, we checked the FGDs’ themes and categories to ascertain the new information obtained from the individual interviews. This contributed to an enhanced understanding of the participants’ perspectives on HIV vaccine trial participation. Representative ideas and quotes from all IDIs were identified for each FGD theme and category. A new category emerged from IDIs and was reported in addition to the FGDs’ themes. The whole text was translated into English. The translation of the text was conducted according to Brislin^39^.

### Ethical Consideration

Ethical clearance was obtained from the Institutional Review Board at Muhimbili University of Health and Allied Sciences (MUHAS) with Ref. No. 2017-06-028/AEC/Vol.XII/85. A permission letter was obtained from the Executive Director of Muhimbili National Hospital (MNH). The first author reviewed the informed consent form and explained to the potential participants the principles of voluntary participation, anonymity, and the right to withdraw from the study at any time without losing any benefit from the health services at the clinic. Written consent was obtained from all participants before data collection. All potential participants consented to the audio-recording during the discussions and interviews. We ensured the anonymity of the information provided by using codes instead of their names in all documents. Participants were reimbursed Tshs 4,000 (equivalent to 1.76 USD) for transportation and their time.

## FINDINGS

### Characteristics of Participants

28 participants participated in the study as follows: 18 participated in FGDs only, 6 participated in IDIs only and 4 participated in both FGDs and IDIs. The following section reports the socio-demographic characteristics of the FGDs and IDIs’ participants separately.

#### Focus group discussion

The ages of the 22 participants ranged from 19 to 50 years, with a mean age of 37.2 (SD=7.8). Of these 22 participants, 16 were males. Most of the participants had primary education levels. 10 of the participants were self-employed, performing activities that enabled them to obtain an income. 18 of the participants were single (Table 2a).

**TABLE 2a:** Socio-demographic characteristics of the participants in FGDs

Characteristic	Numbers
**Age (Years)**	
18–27	4
28–37	10
38–47	5
48 and above	3
**Total**	**22**
**Gender**	
Male	16
Female	6
**Total**	**22**
**Level of education**	
Primary	15
Secondary	5
College	2
**Total**	**22**
**Occupation**	
Employed	3
Unemployed	9
Self-employed	10
**Total**	**22**
**Marital status**	
Single	18
Married	4
**Total**	**22**

#### In-depth interview:

The ages of the 10 participants ranged from 25 to 44 years, with a mean age of 32.6 (SD=5.8). Of these 10 participants, 6 were males. Most of the participants had primary education and half were self-employed. 8 participants were single (Table 2b).

**TABLE 2b:** In Depth Interviews

Characteristic	Numbers (also in FDG)
**Age (Years)**	
18–27	2 (1)
28–37	3 (2)
38–47	5 (1)
48 and above	0 (0)
**Total**	**10**
**Gender**	
Male	6 (3)
Female	4 (1)
**Total**	**10**
**Level of education**	
Primary	7 (3)
Secondary	3 (1)
College	0 (0)
**Total**	**10**
**Occupation**	
Employed	2 (1)
Unemployed	3 (2)
Self-employed	5 (1)
**Total**	**10**
**Marital status**	
Single	8 (4)
Married	2 (0)
**Total**	**10**

### Themes and Categories

Motivations and barriers to participating in HIV vaccine trials were the 2 themes identified in this study. Both themes were derived from the FGDs. 3 categories are reported as motivators and 3 categories as barriers. Among the 6 categories from the themes, 5 categories were derived from FGDs and IDIs while 1 category emanated from IDIs only. The findings are presented together for both FGDs and IDIs (Table 3).

**TABLE 3:** Summary of Themes and Categories

Themes		Motivations to participate in HIV vaccine trials		Barriers to participation in HIV vaccine trials		
Categories	Altruism	Desire to reduce the risk of HIV infection	Social support	Fear of the vaccines' side effects	lack of information about HIV vaccine	HIV related stigma among vaccine trial participants

### Motivation to Participate in HIV Vaccine Trials

Participants reported different factors that would drive them to participate in the trials. Altruism, the desire to reduce the risk of HIV infection, and social support were the main motivators for IDUs to participate in HIV vaccine trials as described in the following;

### Altruism

Participants expressed a desire to participate in HIV vaccine trials with the hope that a successful vaccine would benefit many people in Tanzania and other countries. Also, they hoped that their participation would not only result in an effective HIV vaccine but might also encourage others to be vaccinated against HIV infection as expressed below:

*“When I decide to participate in a vaccine trial against HIV infection, I will be helping my country. Also, if this vaccine becomes effective, it will be helpful to me, as well as other people and other countries.”* (FGD2, participant 14, male, age 40)

Some participants specifically expressed an eagerness to see an effective vaccine developed. They were interested to find out if the vaccine might be discovered because of their efforts. They stated that it would be difficult to get positive results from the vaccine unless they volunteer. The participants in this study also expressed that their participation in the trials was a motivating factor because it might lead to the development of an effective HIV vaccine that could be widely used in the country:

*“No one knows if the vaccine works. How can we know? So, we need to volunteer for the study to get an effective HIV vaccine that will be useful for other people in our country.”* (FGD2, participant 10, male, age 46)

Participants also expressed personal interests in receiving an alternative treatment and a possible cure for HIV infection. They reported that Antiretroviral drugs (ARVs) do not cure the disease; rather alleviate the severity of HIV infections. This awareness prompted them to understand the importance of participating in HIV vaccine trials as stated below:

*“I have heard about it, and until now there is no cure. I am motivated to participate to develop a treatment that will help other people.”* (FGD1 participant 2, male, age 48)

Other participants expressed the fact that they would be delighted to tell community members about the benefits of participation in HIV vaccine trials. They believed that if their community could see the results of their participation, it might influence others to participate in future vaccine trials study as expressed below:

*“If we reach the community, we will inform them that we were involved in the vaccine trials and that the trials have provided the best answers; the trial has been achieved, and its advantages are the same as you see us. These trials are both positive and harmless, and we volunteered as pioneers.”* (FGD2 participant 12, male, age 38)

Participants expressed interest in being in a vaccine trial to obtain the positive results of a potential vaccine, knowing that they contributed to its development. Their main satisfaction was to know that other people might also be motivated to join the study. This factor was one of the strategies they thought they could use to attract their colleagues as described below:

*“If the vaccine brings meaningful results, it will be a good example for other people who will see that we have contributed to the vaccine until the vaccine becomes available.”* (FGD2, participant 9, male, age 50)

Participants in the IDI had similar views on motivation to participate in an HIV vaccine trial through altruism. The information provided by individual interviews corroborates with that from FGDs. Participants stated that the experience of living with their relative infected with HIV drove them to participate in the trial. They expressed a desire to fight HIV/AIDS through the development of a preventive HIV vaccine. One participant remarked:

*“… I have been hurt to see that some of my family have been affected by HIV disease, so when I hear that there is vaccine trial, I am glad that at least it can save the family and other people who have survived”* (IDI, participant 1, Female, age 27).

### Desire to Reduce the Risk of HIV Infection

Participants expressed that they would participate in HIV vaccine efficacy trials to reduce their risk of HIV infection. They described that education that will be provided during the HIV vaccine trial would help them to recognise their risk behaviours and health status. Health screening would help to know their status thus protecting themselves from HIV infection as described below:

*“I am ready to be enrolled in a study because this activity involves participant education of risk-related behaviour, including screening for different infectious diseases. Participating in HIV vaccine trials will give information that helps to protect me from infection”* (FGD2, participant 15, male, age 35).

Participants verbalised that they were motivated to participate in HIV vaccine trials to prevent infection among the at-risk population. They stated that it was a common practice to share needles and syringes, especially because of drug shortages and subsequent cravings. Some participants expressed how painful it was to remember some of the risky practices they had previously engaged in. This painful memory motivated them to participate in HIV vaccine trials. One participant stated:

*“What happens is that we share the drug using the same syringe and needle for all of us so that everyone will have the drug to treat the addiction. You will be forced to trust this person even if you do not know his HIV status. This pains me a lot when it comes to my mind. To me, I will be willing to participate in HIV vaccine trials to facilitate the availability of prevention of HIV infection”* (FGD 1, participant 1, male, age 50)

For IDIs, the desire to reduce the risk of HIV infection as a motivator to participate in the vaccine trial was expressed in the aspects of individual sexual behaviour and experience from harm reduction programs, which were not revealed in FGDs. In this case, participants verbalised that HIV/AIDS is a pandemic disease that is prevalent throughout the country. They expressed the hope that participation in an HIV vaccine trial is an important way to make a vaccine available and thereby preventing HIV infection. The availability of an HIV vaccine might reduce the possibility of at-risk groups contracting HIV, One of the participants explained:

*“HIV infection is a national catastrophe because you can protect yourself from infection, but you can get infected from others. Young people sometimes stay longer [without sex], when they get it [sex], they become confused and therefore forget to use a condom, which may lead to HIV infection. To me, I think, participating in the trials will facilitate the development of the vaccine and thus reduce HIV infection in the community.”* (IDI, participant 9, male, age 33)

Another IDIs’ participant commented that exposure to different harm reduction programs such as the Syringe Exchange Program (NSEP) might have improved their awareness of other health-promoting activities. Effective education provided by Non-Government Organisation (NGOs) before joining the methadone clinic increased the participants’ motivation to participate in HIV vaccine trials as exemplified by the following statement:

*“I was involved in the MDF program [one of the NGOs dealing with SEP]; they were educating us on how we can reduce our risk of HIV infection. To me, participation in an HIV vaccine trial is not a problem because I already know how the MDF works.”* (IDI, participant 4, male, age 37)

### Social Support

Participants in this study reported that social support was an essential motivating factor to participate in HIV vaccine trials. This was based on opinions from close people such as family members, friends, or sexual partners. They verbalised that informing the people they trusted was important because they could provide support and guidance throughout the trial period. They remarked that psychosocial support provided by family members could be a crucial aspect for them to participate in HIV vaccine trials. One of the participants said:

*“…Family members are important people to be involved in decision making toward participation in an HIV vaccine trial. In case the trials bring adverse effects, they will be in the forefront line for guiding and advising on how to handle the problem.”* (FGD3, participant 6, female, age 19)

Another participant expressed the following:

*“Involving someone is important to me so that he can assist with counselling because when you discuss with the person, he will help in advising about whether the thing you want to do is good or not.”* (FGD2, participant 11, male, age 40)

Some participants expressed that they would only follow the family/friends/loved one's opinions, which are congruent with the participants’ own intention to participate. They also added that if the close relative disagrees with their opinions of participating in the HIV vaccine trial, then they would provide more information. This was said to help the family member to understand the participant's needs as stated below:

*“For me to participate in an HIV vaccine trial, I will need to involve my close relatives. I know they* [family members] *cannot refuse. If they refuse, then I will not force them. Instead, I will inform them about the HIV vaccine efficacy trials until they understand”* (FGD3 participant 5, female, age 37)

For the social support as the motivating factors, participants in IDIs expressed similar findings. This validates the information provided in the FGDs. Participants verbalised different reasons for involving family members, including avoiding blame when something bad happens, and that family members were not informed.

*“I would like to involve the family because if something bad happens, the family may ask you, why didn't you tell us? So, it's good to involve your closest people.”* (IDI participant 4, male, age 37)

### Barriers to participation in HIV vaccine efficacy trials

Participants expressed several factors that would hinder them from participating in an HIV vaccine trial. Perceived fear of vaccine side effects, lack of information about HIV vaccine studies, and HIV related stigma towards participants were the main factors that would demotivate participation in HIV vaccine trials.

### Perceived fears of the vaccine side effects

Participants were worried about the effect of the vaccine on their bodies. They mentioned different perceptions related to the effectiveness of the vaccine. They asserted that the side effects of the vaccine might be difficult to handle. Lack of evidence from people in their community who had participated in previous HIV vaccine trials increased the fear of participation as stated below.

*“To me, participation in a vaccine trial is very difficult. It would seem as if I am endangering my life for being vaccinated with an experimental vaccine”* (FGD2, participant 13, male, age 45).

Participants mentioned that people who believe that HIV vaccine contents are harmful could discourage one from HIV vaccine participation. They were worried that those who would volunteer to receive the vaccine might die because of the vaccine materials injected into their body as stated below:

*“If you try to involve other people such as relatives, they can tell you a completely different story. People may say, ‘you are going to be the first person to be harmed by the vaccine. the drug is going to be tested on you, you can die.’ You do not know what effects the vaccines have on your body”* (FGD1, Participant 8, male, age 29).

In the case of perceived fears of the vaccine side effects, the findings from IDIs are similar to those reported in FGDs. This validates the overall perception of the experimental vaccine among the participants. Additionally, the IDI participants were concerned about the safety of the vaccine. They were not sure of the ingredients in the vaccine and thus feared the effects that might occur as a result of an experimental vaccine:

*“I would like my safety to be protected because anything done in the experimental vaccine means that it hasn't been proven 100 percent safe. So, when I volunteer in the HIV vaccine trial, how will my safety be guaranteed? What if it fails? So, I have to doubt anything that is in the test because it is not directly said to provide immunity”* (IDI, Participant 4, male, age 37).

### Lack of information about HIV vaccine studies

Participants expressed concern about the lack of knowledge about HIV vaccine trials. Lack of information was a hindrance to participants to volunteer in a vaccine trial if one was available. Some participants stated that they had heard about an HIV vaccine trial before this study, but did not understand what it was all about. This lack of knowledge discouraged them from participating in the trial. They asserted that they would allow to be recruited if they understood more about the nature of the vaccine and how it works. They expressed:

*“I had heard in the media about HIV vaccines, but I did not fully understand where these vaccines come from. If I get enough information on this, it will be easy for me to be involved in an HIV vaccine study”* (FGD1Participant 2, male, age 48):

Another participant added:

*“I cannot get involved in an activity if I don't understand what the activity is about. Education should be the priority because when a person is knowledgeable, he/she may help to convince and motivate others to join the study”* (FGD 2 participant 12, age 38).

Other participants argued that drug users had difficulty understanding information about HIV vaccine trials. They referred to the hardship experienced during the recruitment of participants for the methadone clinic. They reported that drug users could not understand the importance of methadone and continued to inject illicit drugs even after educating them. One peer educator who used to recruit drug users from the street and educate them about the importance of attending the clinic and using the methadone treatment remarked:

*“We followed and educated them about the importance of using methadone at the clinic. We told them that the drug is free but they were so difficult to understand. …I'm not sure if they can understand and be motivated to engage in the HIV vaccine trial.”* (FGD3, participant 7, female, age 24)

Another participant opposed the ideas and added:

*“…we drug users are not a problem but when awareness is given, then people will understand. What is needed is just information and education. The only thing we ask for is education. People will be motivated to participate if given appropriate education”* (FGD3 Participant 6, Female, age 19).

### Stigma towards HIV vaccine trial participants

Stigma towards HIV vaccine trial participation was prominent during IDIs. This concept did not emerge during the FGDs. In IDIs, many participants reported fears of being labelled and criticised by community members and people around them, including relatives. They stated that people might point fingers at them if they participate in the HIV vaccine efficacy trial because they believed that the participants are infected with HIV, as verbalised by one of the participants below.

*“…so, everyone will be pointing his/her finger at you because you participated in the HIV vaccine trials. The community can discriminate against you because they think you already have HIV, which has no treatment”* (IDI, participant 1, female, age 27)

Other participants were worried about social isolation in the community when participating in the HIV vaccine trial. They asserted that the community members would shun and even isolate them if the vaccine would not be effective:

*“I would like to volunteer to participate and be given the vaccine material to see if it works. However, if I get the vaccine material and it does not work, this will hurt me. …and the community will look at me negatively and even isolate me.”* (IDI, participant 1, female, age 36)

Overall, the participants reported HIV related stigma towards HIV vaccine trial participants as an obstacle that needed to be eliminated through community involvement and education. They expressed that community sensitisation using various education materials such as fliers could help to promote community awareness and thus decreasing stigma towards HIV vaccine trial participants.

### Comparison of findings from FGDs and IDIs

The findings from FGDs and IDIs correspond to each other in the following aspects: Participants from both FGDs and IDIs expressed altruism, desire to reduce HIV infection, and social support as motivations to take part in HIV vaccine studies. In addition, the 2 methods revealed that the perceived fear of vaccine side effects would hinder participation in HIV vaccine studies. While the lack of information about HIV vaccine studies became prominent in FGDs, during IDIs, stigma towards HIV vaccine trial participants emerged as an additional hindrance towards participation in HIV vaccine studies. Also, the participants who were reluctant to share their views in FGDs provided useful information during the individual interviews. Thus, the findings from IDIs complement those from FGDs in a meaningful way.

## DISCUSSION

This study highlights the important factors that may motivate IDUs to participate in HIV vaccine trials. Various factors that may prevent them from participating in HIV vaccine trials are reported as barriers. In the current study, participants are motivated to participate in HIV vaccine trials through altruism, a desire to reduce the risk of HIV infection, and social support. In contrast, perceived fear of vaccine side effects, lack of information about HIV vaccine trials, and HIV related stigma towards participants are the barriers to participation in the hypothetical HIV vaccine trial.

### Motivation to participate in HIV vaccine trials

Based on our findings, altruism is an important motivating factor for participation in HIV vaccine efficacy trials. Participants in this study had experienced people affected by HIV/AIDS. This experience may be a driving force to participate in an HIV vaccine trial. In other studies in the same setting, altruism was reported to be the primary motivator for participants to participate in the HIV vaccine trial.^19,40^ This indicates that altruistic reasoning plays an essential role in motivating participants to join HIV vaccine trials in Tanzania. Similar reasoning is also reported in Kenya whereby the willingness to participate in HIV vaccine efficacy trials was driven by various forms of altruism.^41^ Further evidence to support altruism as the motivating factor has been reported in the USA, the Netherland, and Canada^42,43^. Given the participants’ responses in this study, our findings suggest that the partcipants’ lives might have meaning and purpose because of participation in an HIV vaccine trial, particularly if it yields a positive outcome.

The desire to reduce the risk of HIV infection as a motivator for participation in HIV vaccine trials can be attributed to the high-risk behaviours that participants had experienced before joining the study. The participants are greatly affected by their memories of sharing contaminated needles/syringes and unsafe sexual behaviours as described in the Health Belief Model.^44,45^

This may have motivated them to participate in HIV vaccine trials to reduce HIV infection among themselves and the community at large. A multi-site study in the US, Canada, and the Netherlands revealed similar findings that volunteers were motivated to participate in the HIV vaccine trial to reduce risk behaviour.^22^ Such findings were also reported in Philadelphia where protection from HIV infection was the motivator to participate in the HIV vaccine trial.^46^ The desire to reduce the risk of HIV infection may also be accounted for by the knowledge of harm reduction program that participants were involved in before the current study.^12,28^ Thus, participants perceived HIV vaccine trials as one of such programs for HIV risk behaviours reduction. Therefore, intensive training is needed to differentiate between HIV vaccine trials and other risk behaviour reduction programs during the implementation of actual vaccine trials. Likewise, the motivation to participate in an HIV vaccine trial for reducing HIV infection was reported in a phase I/II HIV vaccine trials study among police officers.^40^ Given the experience of participants in our study, motivation to participate in vaccine trials to reduce high-risk behaviours is an important factor to consider when planning for future HIV vaccine trials among IDUs.

In the context of social support, IDUs demonstrate the key abilities needed to make meaningful decisions about HIV vaccine trial participation. Similarly, previous HIV vaccine studies in Tanzania show that social support plays an essential role in HIV vaccine trials.^18,47^ In Tanzania, the reported importance of involving close people when making decisions may be described by the socio-cultural experience of household decisions among couples^48^ and the type of family patterns.^49^ This is similar to the study conducted in the United States which reported consultation with other people was one of the factors in the decision-making process among adolescents.^50^ This reinforces our understanding that information sharing is important for informed decision-making. It also implies that participants have a meaningful relationship with other people and value their input when making difficult decisions. The findings in our study also correlate with findings from South Africa, whereby the ultimate decision to engage children in HIV vaccine trial participation rested on their mothers after they had shared information with their significant others.^51^ However, further research is needed in this area to explore the social and behavioural characteristics of IDUs who can be motivated to participate in HIV vaccine trials based on consensus from significant others.

### Barriers to participating in HIV vaccine trials

The reported fears of vaccine side effects as a barrier to participation in HIV vaccine trials may be contributed by the lack of proper information about the nature of vaccine materials. Similarly, the phase I/II HIV vaccine trials among police officers in Dar es Salaam, Tanzania, reported fears of vaccine side effects as one of the reasons to decline from participating in an HIV vaccine trial.^52^ Likewise, a study in India showed that participants feared vaccine-induced HIV infection.^53^ Thorough and accurate information related to the vaccine is needed for potential HIV vaccine trial participants. Expanding education about HIV vaccine trials may help to decrease misperception and misinformation. Promoting awareness and comprehensive education for participants about what to expect during the trial is crucial for effective HIV vaccine trial participation.

Lack of information about HIV vaccine trials can be described by the fact that research findings have not been adequately disseminated among the population of interest. Dissemination of HIV vaccine-related information is important for raising awareness in the participating community.^54^ A previous study in Uganda reported improved communication between participants and research staff that created a sense of community ownership among participants.^55^ Nevertheless, a study among transwomen in 4 cities of the USA revealed that having either no exposure or limited exposure towards HIV vaccine trials which was translated as receiving inaccurate information from the laypeople^56^ is a barrier to participation. The findings in our study indicate that the recruitment of prospective participants in an HIV vaccine efficacy trial requires sufficient education to address misperceptions. Such education may potentially decrease barriers towards participation in the vaccine trials. In other words, for effective HIV vaccine efficacy trial participation among IDUs, participants must have a broader understanding of the nature and procedures of the HIV vaccine trials.

As revealed in the present study, HIV-related stigma may prevent participants from volunteering for HIV vaccine efficacy trials. The negative reactions from their communities have greater impacts on the decision to participate. Such negative reactions and their impacts on participation in HIV vaccine studies have also been reported in Kenya.^57^ Participants in our study believed that their participation in an HIV vaccine trial would expose them to prejudicial and discriminatory practices similar to those directed at HIV positive people. Several studies have reported similar findings in other countries.^58–61^ These barriers may be reduced by providing the correct information about the HIV vaccine program. In HIV vaccine efficacy trials, high-risk populations are required for participation. Based on the findings of our study, IDUs represent a good vaccine trial population, as they have been involved in many health promotion programs. Researchers must provide educational materials and ensure that all behavioural and social needs are met before, during, and after the vaccine trials.

### Limitation

This study is not without limitations. First, the study sample was recruited from the methadone clinic which might be different from IDUs in the general population. However, the risk and behavioural characteristics of the participants validate the information. The findings of 0ur study are valuable for planning future HIV vaccine efficacy trials. Second, although the findings of this study should not be generalised beyond the studied sample, the information obtained is important when formulating an HIV research study in a similar setting.

The use of the qualitative method allowed the authors to examine a study sample that had not been previously investigated in Tanzania. Finally, the integration of FGDs and IDIs data as a form of triangulation has been challenged in establishing rigour^62,63^ and therefore might have affected the integrity of findings. However, the current study used data from both methods to complement each other. For example, the 4 participants who were reluctant to express their ideas during FGDs appeared more interactive during the IDIs.

Complementing is important to the qualitative inquiry as it allows for the recognition of multiple realities. In this case, the IDIs added additional information that was not recognised in the FGDs. The combination of 2 sources of data increased the richness of the information obtained, thus making the findings more valuable.

## CONCLUSIONS

Participation in a hypothetical HIV vaccine trial among IDUs is influenced by positive and negative factors. Actual recruitment plans could be made through a better explanation of HIV vaccine trials, the expected individual and collective benefits associated with the trials. Correct information about the HIV vaccine studies and community sensitisation is likely to enhance participation in future HIV vaccine trials.
